# The Collection of Rhizosphere Microorganisms: its importance for the study of associative plant-bacterium interactions

**DOI:** 10.18699/VJ20.623

**Published:** 2020-05

**Authors:** O.V. Turkovskaya, S.N. Golubev

**Affiliations:** Institute of Biochemistry and Physiology of Plants and Microorganisms of the Russian Academy of Sciences, Saratov, Russia; Institute of Biochemistry and Physiology of Plants and Microorganisms of the Russian Academy of Sciences, Saratov, Russia

**Keywords:** microbial culture collection, Azospirillum, rhizosphere, plant-growth-promoting rhizobacteria, associative symbiosis, коллекция микроорганизмов, Azospirillum, ризосфера, стимулирующие рост растений ризобактерии, ассоциативный симбиоз

## Abstract

Microbial culture collections are very important components of biological science. They provide researchers with material for studies and preserve biological resources. One such collection is the Collection of Rhizosphere Microorganisms, kept at the Institute of Biochemistry and Physiology of Plants and Microorganisms of the Russian Academy of Sciences, Saratov (IBPPM). Its activity is primarily directed toward the isolation and preservation of microorganisms from the plant root zone. The international research interest in microorganisms from this ecological niche is not waning, because they are very important for plant growth and development and, consequently, for plant breeding. The group of bacteria with properties of significance for plants has been given the name “plant-growth-promoting rhizobacteria” (PGPR). This group includes nitrogen-fixing soil alpha-proteobacteria of the genus Azospirillum, which form the core of the IBPPM collection. First discovered by Brazilian scientists in the 1970s, azospirilla are now a universally recognized model object for studying the molecular mechanisms underlying plant-bacterium interactions. The broad range of useful properties found in these microorganisms, including the fixation of atmospheric nitrogen, production of phytohormones, solubilization of phosphates, control of pathogens, and formation of induced systemic resistance in the colonized plants, make these bacteria an all-purpose tool that has been used for several decades in basic and applied research. This article reviews the current state of Azospirillum research, with emphasis on the results obtained at the IBPPM. Scientific expeditions across the Saratov region undertaken by IBPPM microbiologists in the early 1980s formed the basis for the unique collection of members of this bacterial taxon. Currently, the collection has more than 160 Azospirillum strains and is one of the largest collections in Europe. The research conducted at the IBPPM is centered mostly on the Azospirillum structures involved in associative symbiosis with plants, primarily extracellular polysaccharide-containing complexes and lectins. The development of immunochemical methods contributed much to our understanding of the overall organization of the surface of rhizosphere bacteria. The extensive studies of the Azospirillum genome largely deepened our understanding of the role of the aforesaid bacterial structures, motility, and biofilms in the colonization of host plant roots. Of interest are also applied studies focusing on agricultural and environmental technologies and on the “green” synthesis of Au, Ag, and Se nanoparticles. The Collection of Rhizosphere Microorganisms continues to grow, being continually supplemented with newly isolated strains. The data presented in this article show the great importance of specialized microbial culture repositories, such as the IBPPM collection, for the development and maintenance of the microbial research base and for the effective solution of basic and applied tasks in microbiology.

## Abstract (ru)

Коллекции микроорганизмов – один из важнейших компонентов биологической науки, кото- рый обеспечивает исследователей необходимым материалом и сохраняет биологические ресурсы. Тако- вой является Коллекция ризосферных микроорганизмов Института биохимии и физиологии растений и микроорганизмов Российской академии наук (ИБФРМ РАН), деятельность которой сосредоточена в первую очередь на выделении и сохранении микроорганизмов, выделенных из корневой зоны растений. Инте- рес мировой науки к микроорганизмам этой экологической ниши не ослабевает по причине их большой значимости для роста и развития растений и, следовательно, для растениеводства. Группа бактерий, об- ладающих полезными для растений свойствами, получила название PGPR (plant growth promoting rhizobacteria – стимулирующие рост растений ризобактерии). К ним относятся и почвенные азотфиксирующие альфа-протеобактерии рода Azospirillum, составляющие ядро вышеназванной коллекции. Азоспириллы, открытые в 70-х гг. прошлого века бразильскими учеными, в настоящее время являются признанными во всем мире модельными объектами для изучения молекулярных механизмов растительно-микробных взаи- модействий. Фиксация атмосферного азота, продукция фитогормонов, солюбилизация фосфатов, контроль патогенов, формирование у растений индуцированной системной устойчивости – целый комплекс полез- ных свойств делает их универсальным инструментом для фундаментальных исследований и практического применения. В обзоре обсуждается современное состояние исследований по бактериям рода Azospirillum с акцентом на результатах, полученных коллективом ИБФРМ РАН (г. Саратов). Экспедиции по Саратовской об- ласти, проведенные микробиологами института в начале 1980-х гг., заложили основу уникального собрания представителей этого бактериального таксона, которое сегодня включает более 160 штаммов и считается одним из самых крупных в Европе. Исследования сотрудников ИБФРМ РАН преимущественно сосредоточе- ны на структурах азоспирилл, вовлеченных в образование ассоциативного симбиоза с растениями. Прежде всего это внеклеточные полисахаридсодержащие комплексы и лектины. Развитие методов иммунохимии во многом позволило выяснить, как в целом организована поверхность бактерий. Благодаря изучению ге- нома азоспирилл существенно углубилось понимание роли вышеупомянутых структур, а также подвижно- сти бактерий и формирования ими биопленок при заселении корней. В прикладном аспекте заслуживают внимания исследования азоспирилл, направленные на развитие агро- и экотехнологий, а также технологии «зеленого» синтеза наночастиц золота, серебра и селена. Коллекция продолжает развиваться, пополняясь новыми штаммами, показывая большое значение специализированных собраний микроорганизмов для создания и поддержания исследовательской базы и эффективного решения фундаментальных и приклад- ных задач микробиологии.

## Introduction

The conservation and development of biological collections in the Russian Federation is part of the interdepartmental and interdisciplinary priority problem of preserving biological resources and biodiversity, as well as the national biological and food security building. The solution of this problem is the basis for the sustainable development of Russian science as a whole, modern science-intensive industries, and training of qualified personnel (Recommendations of the “round table”…, 2011). The currently existing collections (according to the World Federation for Culture Collections (WFCC), there are 715 of them (http://www.wfcc.info/ccinfo)) are tentatively divided into three categories (Kalakutskii et al., 1996; Ivshina, 2012): nonspecialized collections (service, integrated, and public), specialized collections (for the study and preservation of microorganisms of specific groups for specific purposes), and research collections (private, highly specialized). Until recently, a very large number of Russian collections were on the verge of ceasing their activities owing to the lack of funding. In recent years, with the approval of the Plan for the Development of Biotechnologies and Genetic Engineering (Decree of the Government of the Russian Federation, 2013), an attempt was made to provide targeted organizational and financial support to intensely working collections.

The above Plan includes the foundation of large bioresource centers (BRCs), which will be the most important element for the development of biotechnology in Russia (Kalakutsky, Ozerskaya, 2011), and integration into the European and global information networks of biological resources. It should be noted that there are already many BRCs in the world, which are represented by both departmental and private non-profit organizations. One such BRC was established in 2014 on the basis of a leading Russian collection (the Russian Collection of Industrial Microorganisms, VKPM), designed to become the basis of the infrastructure in the field of microbial genetic resources for biotechnological purposes necessary for supporting research in living systems (http://www.genetika.ru/vkpm). Several large Russian collections may claim the BRC status. Most of the collections existing as specialized units at scientific and educational organizations cannot meet the criteria of such large structures, but they deserve attention as well. As a rule, they possess both a significant panel of cultures and the most complete information about them, which is their significant advantage. One such specialized unit is the IBPPM Collection of Rhizospheric Microorganisms (CRM IBPPM).

## The IBPPM Collection of Rhizospheric Microorganisms

The Institute of Biochemistry and Physiology of Plants and Microorganisms, Academy of Sciences of the USSR (IBPPM), founded in Saratov in 1980, was focused on the tasks of increasing crop yields in the Volga region. One of them was the justification of the possibility of using microbial fertilizers and, in this regard, the clarification of the role of associative nitrogen-fixing microorganisms in the nitrogen nutrition of crops. To solve this problem, the diazotrophic bacterium Azospirillum was chosen as a model research object, publications about which had appeared shortly before (Tarrand et al., 1978). The Institute’s microbiologists mastered the methods of isolation and identification of these microorganisms and worked out methods for their conservation. In the course of expeditions in the Saratov region, undertaken to obtain a representative collection of Azospirillum strains associated with wild and cultivated cereals (wheat, rye, oats, millet, maize, sorghum, etc.) (Fedorova et al., 1985; Pozdnyakova et al., 1988), the investigators laid the foundation for a unique set of members of the studied bacterial taxon (see the Figure), becoming part of the IBPPM Collection of Non-Pathogenic Microorganisms.

**Table 1.**
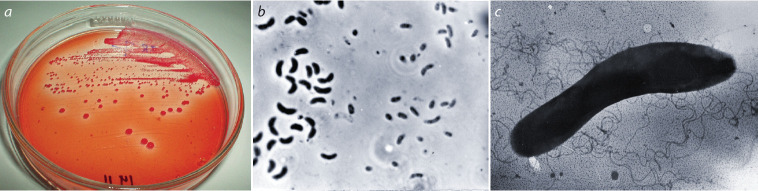
Visualization of Azospilillum bacteria: а, growth of azospirilla on potato agar; b, azospirilla in a liquid medium, 18 h, phase contrast; c, azospirilla under the electron microscope, 18 h, ×25,000, contrasted with uranyl acetate (Pozdnyakova et al., 1988).

The key characteristics of azospirilla in the first stages of isolation were: colony growth on a medium with Congo red (Caceres, 1982) and on potato agar (Tarrand et al., 1978), as well as microscopy and Gram staining. Because the main features of the isolated bacteria were the fixation of atmospheric nitrogen and the promotion of plant growth, the isolates were screened for nitrogenase and IAA-producing activities.

Taxonomic studies were carried out with original strains of azospirilla. In addition to interspecific differentiation by methods based on DNA–DNA hybridization, intraspecific differences were evaluated with two variants of genomic fingerprinting: RFLP and AFLP (Pozdnyakova et al., 1988; Nikiforov et al., 1994a). The plasmid composition of the strains was studied (Matveev et al., 1988). The obtained isolates were screened for restriction endonuclease activity (Nikiforov et al., 1994b). Storage methods were being optimized for four years. Long-term observations showed that Azospirillum strains frozen in liquid nitrogen retained high viability and basic differential properties at –70 °C.

In 2014, the Collection of Non-Pathogenic Microorganisms was transformed into the CRM IBPPM (www.collection. ibppm. ru; http://ckp-rf.ru). It is a specialized scientific collection focused on the gathering and maintenance of nonpathogenic bacteria isolated mainly from the root zone of plants. It is a member of the WFCC with the number 975 and is registered at the World Data Center for Microorganisms (WDCM), No. 1021.

Currently, the collection catalog (www.collection.ibppm. ru) includes about 500 cultures of rhizospheric bacteria belonging to 28 genera: Acidovorax, Aeromonas, Alcaligenes, Aquaspirillum, Arthrobacter, Azospirillum, Bacillus, Bradyrhizobium, Brevundimonas, Comamonas, Ensifer (Sinorhizobium), Enterobacter, Herbaspirillum, Kocuria, Micrococcus, Moraxella, Mycobacterium, Nitrospirillum, Niveispirillum, Nocardioides, Ochrobactrum, Paenibacillus, Pectobacterium, Pseudomonas, Rhizobium, Rhodococcus, Stenotrophomonas, and Xanthomonas. Among them are bacteria isolated from the rhizosphere and rhizoplane of various wild and cultivated plants, endophyte strains, strains associated with aquatic plants (macrophytes), etc.

Members of the genus Azospirillum constitute approximately half of the collection. In addition to the original strains isolated in the Saratov region, the collection includes azospirilla of various geographical origins (Brazil, India, Senegal, the USA, Ecuador, etc.) provided by researchers or other culture collections. In addition, almost all the type strains of known Azospirillum species available in public depositories are maintained. Among the collection strains, there may be members of new species of this genus (Golubev et al., 2018). Thus, the Azospirillum set in the CRM IBPPM is highly representative. For comparison, in DSMZ (Deutcsche Sammlung von Mikroorganismen und Zellkulturen, www.dsmz. de), the total bacterial stock of the genus Azospirillum is represented by 27 strains, of which 6 are type strains; at BCCM/ LMG (Belgian Co-ordinated Collections of Microorganisms/ Laboratory of Microbiology, Faculty of Sciences of Ghent University, bccm.belspo.be), 37 and 10; at JCM (Japan Collection of Microorganisms, jcm.brc.riken .jp), 13 and 5; in ATCC (American Type Culture Collection, www.lgcstandards-atcc. org), 14 and 3, respectively.

## Characterization of the genus Azospirillum

This microorganism was first isolated by Beijerinck in 1923 and described as a “nitrogen-fixing Spirillum”, but the researcher was unable to confirm its ability to fix nitrogen in pure culture and called this microorganism Spirillum lipoferum (Beijerinck, 1925). The genus Azospirillum was rediscovered and described by Tarrand et al. (1978) but became widely known thanks to the scientific enthusiasm and competence of Johanna Döbereiner, who made a significant contribution to the basic and applied research on azospirilla (Döbereiner, Day, 1976; Hartmann, Baldani, 2006).

The taxonomy of the genus Azospirillum, belonging to the family Rhodospirillaceae of the order Rhodospirillales in the class α-Proteobacteria, is developing fast. From the moment the first two species, A. lipoferum and A. brasilense, were described (Tarrand et al., 1978) and until the end of the second millennium, only four more species were discovered: A. amazonense (Magalhaes et al., 1983), A. halopraeferens (Reinhold et al., 1987), A. irakense (Khammas et al., 1989) and A. largimobile (Dekhil et al., 1997). However, since 2000, 16 new species have been described with valid published names: A. doebereinerae (Eckert et al., 2001), A. oryzae (Xie, Yokota, 2005), A. melinis (Peng et al., 2006), A. canadense (Mehnaz et al., 2007a), A. zeae (Mehnaz et al., 2007b), A. rugosum (Young et al., 2008), A. picis (Lin et al., 2009), A. thiophilum (Lavrinenko et al., 2010), A. formosense (Lin et al., 2012), A. humicireducens (Zhou et al., 2013), A. fermentarium (Lin et al., 2013), A. soli (Lin et al., 2015), A. agricola (Lin et al., 2016), A. ramasamyi (Anandham et al., 2019), A. griseum (Yang et al., 2019), and A. palustre (Tikhonova et al., 2019). In 2014, Azospirillum irakense was reclassified as Niveispirillum irakense comb. nov. and Azospirillum amazonense, as Nitrospirillum amazonense gen. nov., sp. nov. (Lin et al., 2014). Thus, at the time of this writing, the genus Azospirillum includes 20 species with valid published names. It is important to note that two more species are recognized as members of the genus: A. palatum (Zhou et al., 2009) and A. himalayense (Tyagi, Singh, 2014), whose names were not validly published in conformity with the rules of the International Code of Nomenclature of Bacteria.

The onset of the era of genomic sequencing highlighted the development of reliable criteria for the comparative assessment of the genomes of bacteria and archaea for taxonomy and systematics. Recently, the first results of applying a set of phylogenetic tests in the context of the development of microbe genomic taxonomy were obtained for the group of bacteria of the genus Azospirillum whose genomes were present in the GenBank database (Shchyogolev, 2018). The author revealed the dependence of the assessment of the taxonomic position of strains on the type of full-genome data used related to the core or core and variable components of the pangenome. It was noted that there was no unified system of assigning isolates to one or another species yet. Until the genomic databases are filled to the necessary extent with high-quality material, the so-called “polyphase” approach, based on a combination of phenotypic, chemotaxonomic, and genotypic characteristics, remains the most correct in the taxonomy and systematics of prokaryotes.

Azospirilla, chosen as an object of research in the infancy of IBPPM, turned out to be an excellent model for studying associative plant-microbe interactions. Currently, Azospirillum is one of the universally recognized and widely studied plantgrowth- promoting rhizobacteria (PGPR) (Fukami et al., 2018). Such bacteria play an important role in helping the plant adapt to external influences. In this case, a plant-microbial association (associative symbiosis) with new properties determined by positive interactions between partners is often formed.

The extreme diversity of colonizable plant species is characteristic of azospirilla, which indicates the multitude of ecological strategies implemented by these bacteria, as well as their wide adaptive capabilities (Bashan et al., 2004; Baldani et al., 2014; Pereg et al., 2016). Bacteria adapt to their environments through such abilities as fixation of atmospheric nitrogen; solubilization of phosphates; and production of exopolysaccharides, lectins, phytohormones, siderophores, poly-β-hydroxybutyrate, etc. There is evidence (Fukami et al., 2018) that Azospirillum bacteria are involved in the formation of so-called induced systemic resistance (ISR) in partner plants exposed to biotic stress, as well as induced systemic tolerance (IST) to abiotic stress.

Most Azospirillum species were isolated from the rhizosphere of land plants. So far, only three species were isolated from aquatic biotopes: A. largimobile (Dekhil et al., 1997), A. thiophilum (Lavrinenko et al., 2010), and A. griseum (Yang et al., 2019). Sequencing of the Azospirillum genome showed that this bacterium shifted from aquatic to terrestrial existence at the same time as vascular plants appeared on land: about 400 million years ago (Wisniewski-Dyé et al., 2011). Almost half of the Azospirillum genome was acquired as a result of horizontal gene transfer from other terrestrial bacteria. Most horizontally acquired genes encode functions that are critical to environmental adaptation.

The possession of phytostimulation mechanisms makes azospirilla one of the best inoculants that are employed in various countries to manufacture commercial biological products increasing crop yields: Azo-Green™, Zea-Nit™, Graminante™, BioPower®, etc. (Mehnaz, 2015).

## The key results obtained at the IBPPM RAS in studies of Azospirillum bacteria as model objects

At the IBPPM RAS, the use of Azospirillum strains as model objects was focused mainly on the study of the structures involved in the formation of associative symbiosis and/or having important taxonomic significance. First of all, these are extracellular polysaccharide-containing complexes, which play very important and diverse roles in the formation and successful functioning of plant–microbe associations. Azospirilla produce intricate highly aggregated compounds of polysaccharides (PSs), lipids and proteins, as well as free PSs with molecular weights up to 20 kDa. These compounds are stored in the capsular material and released to the environment (Konnova et al., 1994). The capsular PS-containing components of azospirilla are involved in the adsorption of the bacteria to plant roots. Their ability to induce deformations of root hairs of wheat was shown for the first time (Yegorenkova et al., 2001). Novel information was obtained on the primary structures of repetitive PS units in surface lipopolysaccharides (LPSs) and capsular PSs of more than 40 Azospirillum strains of various origins (Fedonenko et al., 2013, 2015). In several strains isolated from plant roots on different continents, molecular mimicry of bacterial surface glycopolymers owing to the presence of identical or structurally similar repeating units in an O-specific PS (O-PS) was observed. It may be conjectured that this mimicry is associated with the implementation of certain strategies during the formation of associations with plants, possibly owing to the presence of several interaction mechanisms, for example, endo- and ectosymbiosis (Konnova et al., 2008; Fedonenko et al., 2015).

Immunochemical methods are a good tool for the study of bacterial surface structures. Scientists can examine structural details of the main surface antigens of azospirilla to find out how the surface of rhizosphere bacteria is generally organized. LPS is a major antigen on the surface of azospirilla; therefore, the O-PS structure determines the immunochemical specificity of these microorganisms (Matora et al., 2005). With account taken of the immunochemical characteristics of the carbohydrate antigens of Azospirillum bacteria, a biotest system for their serological identification has been developed (Bogatyrev et al., 1992).

With the example of azospirilla, a fundamentally new type of microbial R-S dissociation was described, owing to the redistribution of the contributions of two different (full-fledged) O-PSs to the architecture of the bacterial cell surface, depending on the culture age (Matora et al., 2005). Carbohydrate fragments of the glycosylated flagellin of the polar flagellum from A. brasilense type strain Sp7 were isolated and studied, and their chemical structures were determined (Belyakov et al., 2012). These fragments were immunochemically identical to one of the two O-PS somatic antigens of strain Sp7. With account taken of the results obtained, which indicate the identity of antigenic determinants in capsular PSs, exopolysaccharides, and LPSs of azospirilla, it is reasonable to suggest a common pathway (or several intersecting pathways) for the biosynthesis of carbohydrate surface structures in Azospirillum bacteria.

For the first time, a variant of enzyme-linked immunosorbent assay (ELISA) of microsediments of soil suspensions was proposed that uses antibodies against Azospirillum LPSs. The assay allows the detection of the somatic bacterial antigen in soil. With the optimized ELISA variant, the dynamics of in situ detection of the somatic antigen of A. brasilense associative bacteria introduced into soil was studied (Shirokov et al., 2015).

A prominent direction in the progress of the immunochemical methodology at the IBPPM RAS is the study of unique physicochemical and biochemical properties of gold nanoparticles and their bioconjugates. By using colloid gold as a carrier and an adjuvant, as well as the phage display method, procedures have been developed to prepare antibodies to various antigens and haptens (Matora et al., 2005; Dykman et al., 2010). Gold and gold-silver nanoparticles were conjugated with antibodies to flagellin, LPS, and genus-specific surface-protein determinants of A. brasilense type strain Sp7. Electron microscopic analysis of the A. brasilense Sp245 cell surface involving antibodies labeled with metal nanoparticles revealed flagellin determinants of the polar flagellum, originally shielded from their environment by an LPS sheath in these bacteria (Shirokov et al., 2017).

Significant progress has been made in the genetics of motility, plasmid biology, and the genome organization and dy- namics of Azospirillum bacteria (Katsy, 2011, 2014). A new type of social motility was revealed: spreading in a semiliquid medium with the formation of microcolonies. It is such spreading that is of decisive importance when wheat roots are colonized by azospirilla, while swarming is the dominant mode of social motility under laboratory conditions (Shelud’ko et al., 2010). It was shown that external factors (the presence of certain plant lectins, plant exudates, etc.) and spontaneous and induced changes in the genome, in particular, in the structure of megaplasmids, have a great influence on the social behavior of the bacteria. The genome changes are accompanied by phenotypic variations in the social motility of azospirilla (swarming → accelerated swarming; swarming → spreading with the formation of microcolonies; spreading with the formation of microcolonies → accelerated swarming), and they can lead to changes in the formation of biofilms and in the early stages of plant-root colonization (Katsy, Prilipov, 2009; Schelud’ko et al., 2009).

Insertion elements responsible for the plasticity of A. brasilense megaplasmids were described for the first time. New knowledge was obtained on the primary structures and functions of several such plasmids (Katsy, Prilipov, 2009). The insertion elements ISAzba1 and ISAzba3, which mediate the fusion of the resident plasmid from A. brasilense Sp245 with foreign DNA, contribute to the enrichment of the Azospirillum genome with genetic material. The genome dynamics of A. brasilense has a significant effect on the structure of the bacterial LPSs and their antigenic properties, as well as on the resistance of these bacteria to heavy metals and nitrites (Katsy, Petrova, 2015). The Azospirillum genes that regulate motility, denitrification, and the production of LPSs and flagella have been identified. A collection of A. brasilense mutants, recombinant plasmids, and Escherichia coli strains containing cloned Azospirillum genes was established (Kovtunov et al., 2013).

Another component of the Azospirillum cell surface is carbohydrate-binding proteins, lectins. These are important structures in the system of “recognition” and the establishment of partnerships at the initial stages of associative bacterium– plant relationships. Studies of Azospirillum lectins at the IBPPM RAS began in the second half of the 1980s (Nikitina et al., 2005). It was for the first time that lectins with interstrain differences in carbohydrate specificity were found on the surface of azospirilla isolated from various sources (30 strains). It was revealed that lectins are distributed evenly on the outer membrane of the azospirilla and do not belong to any morphological structures such as pili or flagella (Karpunina et al., 1995). The dependence of lectin activity in bacteria on culturing conditions was found. Conditions unfavorable for culture growth stimulated lectin activity and vice versa. For the first time, the role of Azospirillum lectins associated with the outer membrane in the adhesion of the bacteria to wheat seedling roots was revealed. Bacterial lectins were found to interact with exocomponents, components of the membrane fraction, and root lectins of plant seedlings (Nikitina et al., 1996). Confocal laser scanning microscopy showed that the location of tritium-labeled Azospirillum lectins was confined to the plasma membrane of wheat seedling root cells. At the initial stages of interaction with the roots, lectins can elicit a broad range of biochemical responses that are part of plant signaling systems (Alen’kina et al., 2014).

A dose-dependent effect (inhibiting or promoting) of bacterial lectins on the germination of seeds of higher plants was shown. The regulatory effect of Azospirillum lectins on a number of their own and plant hydrolytic enzymes was revealed (Alen’kina et al., 2006). From the detected interaction of the polysaccharide-containing complexes of azospirilla with intrinsic lectins, as well as with surface-localized agglutinating proteins of other soil microorganisms (bacilli and rhizobia), participation of these extracellular glycopolymers in Azospirillum aggregation and in interbacterial contacts during the formation of soil communities may be inferred.

For the first time, the ability of azospirilla to reduce gold (III) (AuCl– 4) and selenium (IV) (SeO2– 3 ) to the elementary state (Kupryashina et al., 2013; Tugarova et al., 2014a, b) with the formation of nanoparticles was described. A simple scheme of bacterial synthesis of selenium nanoparticles with extracellular localization was proposed (Tugarova et al., 2018).

Various aspects of the azospirilla life are extensively studied by modern instrumental methods, including various spectroscopy options: Mössbauer, IR Fourier, and Raman scattering (Kamnev et al., 2001, 2018; Kamnev, Tugarova, 2017). By using glutamine synthetase isolated from A. brasilense Sp245 cells as an example, the possibility of applying nuclear gamma resonance spectroscopy (57Co nuclei) to the examination of the structural organization of metal cation binding sites in active centers of enzymes was shown (Kamnev, Tugarova, 2017). For the first time in relation to Azospirillum bacteria, the assimilation of iron and the composition and structure of iron-containing cellular components were studied (Kovács et al., 2016), and so were the interaction and metabolic transformations of cobalt ions by A. brasilense cultures (Kamnev, Tugarova, 2017).

The synthesis of poly-β-hydroxybutyrate (PHB) by azospirilla is the most pronounced response to negative effects in these bacteria. For the first time, changes in bacterial PHB accumulation under prolonged exposure to stress and differences between A. brasilense strains Sp7 (epiphyte) and Sp245 (endophyte) in response to heavy metal stress were shown (Kamnev et al., 2018). A reduced PHB content in 6-day old biofilm formed by the flagella-free mutant A. brasilense Sp245.1610, as compared to the wild-type strain Sp245 was shown by Tugarova et al. (2017). The decrease in PHB content may affect the formation and stability of Azospirillum biofilms.

It is known that under adverse living conditions plants gain advantage if the protective rhizosphere associations contain microorganisms that perform a wide range of functions, including plant nutrition, resistance to abiotic stresses, biocontrol (protection against pathogens), and the removal of pollutants from soil (Tikhonovich, Provorov, 2009). The last function is performed by pollutant-degrading microorganisms. Azospirilla, possessing almost all the above properties, are typical representatives of protective rhizosphere associations. Screening of Azospirillum strains from the CRM IBPPM allowed the first detection of oil-oxidizing activity in some of them (Muratova et al., 2005). Use of the A. brasilense SR80- wheat model showed that oil neither interferes with the plantgrowth- promoting activity of the micropartner nor affects the synthesis of bacterial IAA. A. brasilense SR80 showed chemotaxis not only toward root exudates of wheat but also toward crude oil (Muratova et al., 2005). This strain was also resistant to the toxic effects of glyphosate and showed a consistently high level of IAA production in the presence of the herbicide (Kryuchkova et al., 2005). On the basis of these results, a phytoremediation method was developed for hydrocarbon-contaminated soil in which A. brasilense SR80 was used as one of the bacterial cultures for inoculating plants (a mixture of leguminous and cereal seeds) (Patent RU 2403102). A. brasilense Sp245 associated with the roots of wheat seedlings of cv. Saratovskaya 29 can transform inorganic forms of arsenic (arsenite to arsenate); owing to this ability, the bacteria reduce the toxicity of the element (Lyubun et al., 2006).

On the basis of A. zeae strain from the CRM IBPPM, employees of the Bionovatic group of companies developed and produced a biological product named Organit N, aimed at improving the nitrogen nutrition of plants (bionovatic.ru). According to the manufacturer’s recommendations, the preparation is applicable to cereals, legumes, corn, and sugar beet.

The high efficiency of artificial plant-microbial associations established in vitro with the participation of azospirilla was shown in the development of microclonal propagation technologies for plants to improve the quality of planting material of crops and preserve rare plant species that are sources of valuable biologically active compounds (Tkachenko et al., 2015).

## Conclusions

Despite the rather long period of studying Azospirillum bacteria, the interest of the world scientific community in them does not wane; the number of publications dedicated to azospirilla has been steadily growing during the past decade, reaching 250 articles per year (according to www.scholar. google.com). The knowledge gained serves as a basis for expanding research on the variety of bacteria that form associations and symbioses with plants. It is in this direction that the CRM IBPPM develops at present, and in this regard, the role of such specialized collections can hardly be overestimated. As a result of IBPPM participation in the Russian-European project of the 7th Framework Program of the European Union “Banking Rhizosphere Micro-Organisms” (BRIO No. 266106, 2011–2014), some strains of the Collection were included in the pan-European database on rhizospheric microorganisms, designed to support both research on the rhizospheric microbiome and practical biotechnology (Declerk et al., 2015).

It should be noted that the results described above were obtained by modern methods, including those obtained in Russian and international projects: Russian Science Foundation, Russian Foundation for Basic Research, grants of the President of Russia, state contracts within governmental programs, ISTC, INTAS, NATO, FP-7, etc. The obtained data formed the basis for more than 80 candidate’s and doctoral dissertations defended by researchers of the IBPPM. More than 600 articles were published in Russian and international scientific journals. A scientific school was created under the guidance of Professor V.V. Ignatov, D. Sc. (Biol.), Honored Scientist of the Russian Federation. The school was repeatedly supported by grants of the President of the Russian Federation. Several inventions are protected by patents of the Russian Federation. PGPR strains and pollutant-degrading strains have become the subject of intense interest from domestic small businesses.

Thus, the example of the IBPPM Collection shows clearly the significance of such special collections for the fundamental and applied aspects of biological science.

## Conflict of interest

The authors declare no conflict of interest.
